# Probing THz intersubband absorption using Johnson noise thermometry

**DOI:** 10.1515/nanoph-2023-0752

**Published:** 2024-02-05

**Authors:** Changyun Yoo, Mark S. Sherwin, Kenneth W. West, Loren N. Pfeiffer, Jonathan H. Kawamura, Boris S. Karasik

**Affiliations:** Jet Propulsiton Laboratory, California Institute of Technology, Pasadena, CA, USA; University of California, Santa Barbara, CA, USA; Princeton University, Princeton, NJ, USA

**Keywords:** THz intersubband transitions, Johnson noise thermometry, Tunable Antenna-Coupled Intersubband Terahertz (TACIT) mixer

## Abstract

We investigate the THz intersubband absorption behavior of a single 40-nm wide GaAs/AlGaAs square quantum well (QW) using Johnson noise thermometry. In our measurements, the Johnson noise associated with intersubband absorption is measured from the in-plane conduction channel of the QW while its intersubband absorption behavior is being tuned through the independent control of the charge density and the perpendicular DC electric field. Our measurements enable the study of intersubband absorption of a small (∼20,000 and potentially fewer) number of electrons in a single mesoscopic device, as well as direct measurement of the electron heating from intersubband absorption. By measuring the Johnson noise response to monochromatic THz radiation at 2.52 THz and 4.25 THz at 20 K as a function of the DC electric field over a wide range of charge density, we show that the observed Johnson noise behavior correlates well with the expected intersubband absorption of the 40-nm QW. To explain the absorption features of the experimental results, we model the data by calculating the THz coupling efficiency based on the impedance model for intersubband absorption, which qualitatively reproduces the observed Johnson noise behavior well. Based on the temperature calibration of the Johnson noise measured at 2.52 THz, we deduce an increase in the electron temperature Δ*T*_*e*_ of ∼35 K when the maximum absorption of THz power occurs in the device.

## 1 Introduction

Intersubband transitions in semiconductor heterostructures have been a topic of fundamental interest in semiconductor physics [1], [2], [3], [4], [5], [6], [7], [8], and have played a crucial role in the development of various optoelectronic devices [9], [10], [11], [12], [13], [14], [15], [16], [17], [18], [19], [20], [21]. In particular, the fact that the energy spacing between two electronic subbands can be engineered to be in the THz range (1–5 THz) has motivated and led to the successful development of THz emitters and detectors, well-represented by THz quantum-cascade lasers (QCLs) [11], [12], [13], THz quantum-well photodetectors [14], [15], [16], [17], and, more recently, by various novel THz intersubband direct [18] and heterodyne detectors [19].

In our recent work [19], we demonstrated a THz heterodyne detector (mixer) based on intersubband transitions in a modulation-doped, single GaAs/AlGaAs square quantum well (QW) operating at a wide THz frequency range (2.5–4.3 THz). Our device, named as tunable antenna-coupled intersubband terahertz (TACIT) mixer, uses antenna-coupled intersubband transition to efficiently absorb THz radiation in a relatively small active region (typically ∼ 3 μm × 3 μm in size) [22], [23]. Once the THz radiation is absorbed, the excited electrons in the active region quickly thermalize [24], [25], raising the electron temperature of the 2-dimensional electron gas (2DEG) above the lattice temperature. This increase in the electron temperature strongly affects the in-plane mobility of the high-mobility 2DEG [26], resulting in a non-linear increase in the in-plane resistance that can be exploited for sensitive heterodyne detection. The electron energy relaxation in the involved processes can be very fast (<10 ps) [27], [28], [29], resulting in a wide bandwidth (>6 GHz) useful for practical applications of TACIT mixers [19].

One important aspect for TACIT mixers and for various other intersubband devices is the knowledge of intersubband absorption frequency, depending on the operating condition of the device. For example, for TACIT mixers, the detection frequency is determined by the intersubband transition between the ground (*n* = 1) and the first excited (*n* = 2) subbands. While the subband energy spacing is largely engineered with the QW width (for example, 40 nm for ∼2.5 THz), the intersubband absorption frequency can also be tuned *in-situ* over a wide frequency range (2.4–5 THz for the 40-nm QW) through the DC Stark effect by applying a perpendicular DC electric field oriented along the growth direction of the QW [30], [31]. In addition, the intersubband absorption frequency is further affected by the charge density in the QW via collective effects (depolarization shift and exciton shift) and many-body effects on the potential and subband energy (Hartree potential and exchange and correlation energy) [4], [30], [31], [32], [33]. The many-body effects are especially important for wide QWs used for THz devices where the subband energy spacing is smaller, which makes the relevant frequency shifts a significant fraction of the bare subband energy spacing.

Typically, the study of intersubband transition and its various frequency shifts has been investigated optically [1], [2], [3]. In these measurements, QW heterostructures are excited with a broadband THz source, and the transmitted, scattered, or reflected light is analyzed for the intersubband absorption. For higher absorption efficiency, multiple QW (MQW) structures, multi-pixel device elements, and grating structures are often used [30]. The effects of both the DC Stark shifts [31], [34] and depolarization shifts [33], [35] on the intersubband absorption frequency have been extensively studied in this manner.

In this article, we demonstrate that the intersubband absorption behavior of a QW can be investigated in an alternative method, using Johnson noise thermometry. Previously, Johnson noise in intersubband detectors has been studied both theoretically and experimentally in MQW structures, in which the photocurrent (or photovoltage) associated with intersubband absorption occur in the out-of-plane channel of the MQW [36], [37], [38]. By taking advantage of the in-plane read-out structure and the tuning capability of our intersubband device (TACIT detector), we show that the Johnson noise associated with intersubband absorption can be measured directly from the in-plane channel of a single QW while its intersubband absorption behavior is simultaneously being tuned by the independent control of the charge density and the perpendicular DC electric field. In contrast to conventional optical approach, our method uses a continuous-wave (cw) monochromatic THz source to excite intersubband transitions in a single TACIT device, in which a lens-antenna (quasi-optical) coupling scheme is used to concentrate incoming THz electric fields in the small ( ∼3μm×3μm ) active region, allowing the study of intersubband absorption of a small number (∼20,000 with a typical charge density of 2.0 × 10^11^ cm^−2^) of electrons in a single mesoscopic device using a monochromatic THz source. Furthermore, compared to direct and heterodyne detection performed with TACIT detectors in our previous work [19], the Johnson noise measurement can be performed without applying a DC bias in the in-plane channel of the device and can be implemented using a relatively simple microwave setup where readily available high-gain, low-noise microwave amplifiers can be used for sensitive detection of the Johnson noise power at a microwave frequency. In addition, the Johnson noise measurement allows the direct measurement of the increase in the electron temperature Δ*T*_*e*_ resulting from intersubband absorption, which is useful for both fundamental study of intersubband absorption and for practical device engineering of various intersubband devices [39], [40], [41].

To demonstrate our experimental approach, we measured the Johnson noise response of a single TACIT detector to monochromatic THz radiation at 2.52 THz and 4.25 THz. The TACIT detector was based on a single, 40-nm wide GaAs/AlGaAs square QW, and the two THz frequencies were chosen to probe the intersubband absorption when its intersubband absorption behavior is tuned near the flat-band condition (for 2.52 THz) and far away from the flat band (for 4.25 THz). By measuring the DC field dependence of the Johnson noise responses over a range of charge density and by comparing the experimental data with the self-consistently calculated intersubband absorption behavior, we show that the observed Johnson noise responses correlate well with the expected DC Stark-shifted and charge-density dependent intersubband absorption behavior at both frequencies. Based on intersubband absorption parameters extracted from the self-consistent calculation, we simulated the absorption features expected in the Johnson response by calculating the THz optical coupling efficiency using the impedance model for intersubband absorption [22], [42], which reproduces the qualitative features of the data well. Finally, based on the temperature calibration of the Johnson noise response and its dependence on THz power at 2.52 THz, we extract an increase in the electron Δ*T*_*e*_ ∼ 35 K when the maximum absorption of THz power occurs in the device.

## 2 Device and experimental setup

### 2.1 Device design and fabrication

**Figure 1:**
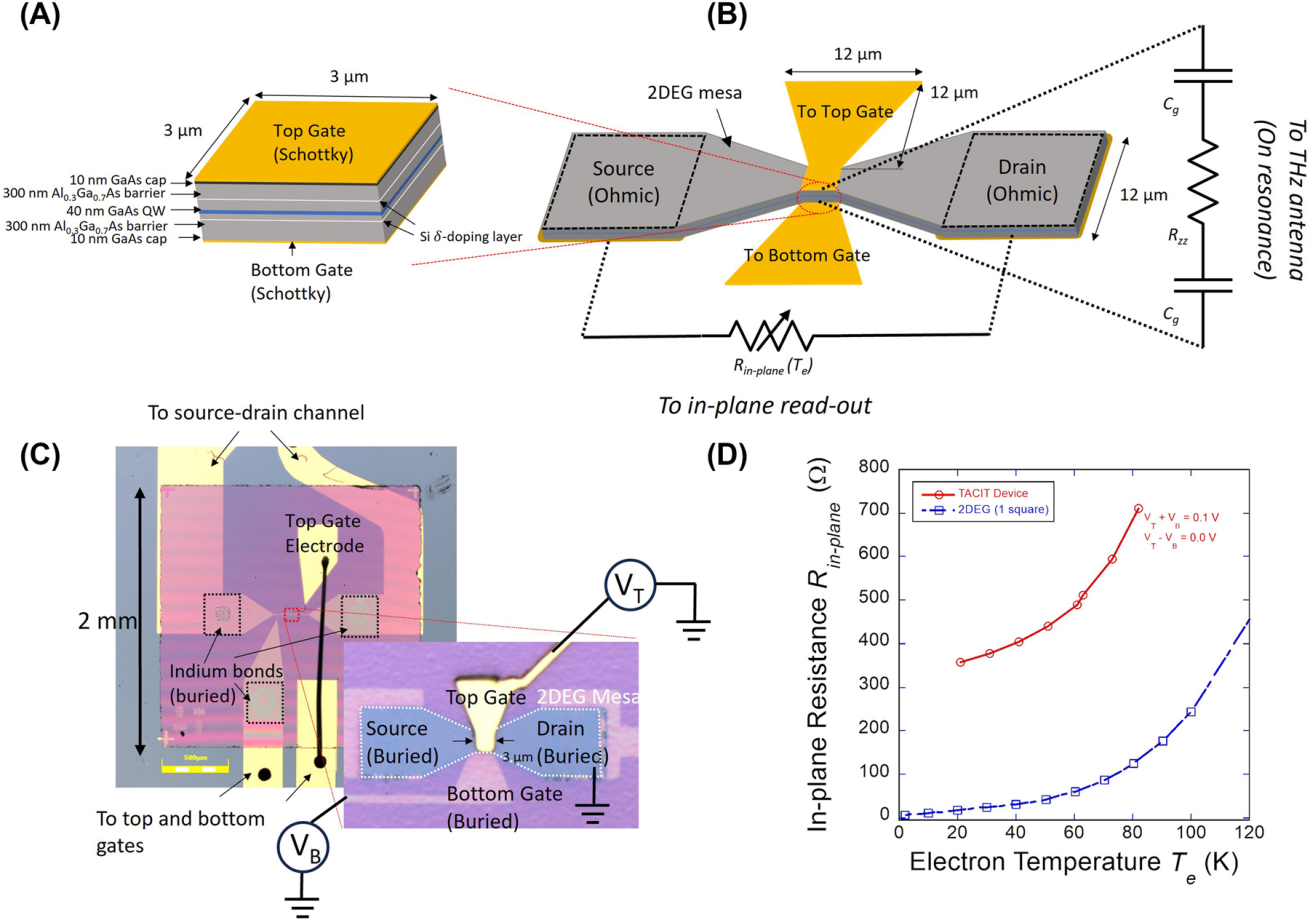
Design, fabrication, and in-plane resistance characterization of tunable antenna-coupled intersubband terahertz (TACIT) detector. (A) Schematic for the QW structure. (B) Schematic showing the bow-tie antenna integrated with the active region and the rest of the 2DEG mesa, as well as the equivalent circuit diagrams for the out-of-plane channel (for THz coupling) and for the in-plane channel (for the Johnson noise read-out) of the device. (C) An optical microscope image showing the fabricated TACIT device. The inset shows the active region of the device. The top and bottom gates are DC-biased with two voltage sources to independently control the charge density and the perpendicular DC electric field applied to the active region. (D) Temperature dependence of *R*_*in*−*plane*_ of the TACIT device (red circle) and the sheet resistance of the 2DEG measured from a Hall bar (blue squares) with a nominal width and length of 40 μm and 400 μm and with a charge density of $∼2.4\times 10^{11}$ cm^−2^.

The active region of the TACIT device is illustrated in Figure 1A. The QW layer was grown with molecular beam epitaxy (MBE) on a 520-μm thick intrinsic GaAs substrate, and consisted of the single, 40-nm GaAs QW layer enclosed on both sides with 300-nm thick Al_0.3_Ga_0.7_As barrier layers and 10-nm GaAs cap layers. The QW was modulation-doped on both sides with Si *δ*-doping layers placed 120-nm away from the center of the QW. The width of the QW and the doping level were chosen to set the 1 → 2 intersubband transition to be near 2.52 THz in the flat-band condition (in the absence of external DC electric field). To enable the device fabrication, the active QW layer was grown on top of a 500-nm thick Al_0.74_Ga_0.26_As etch-stop layer (not shown in the figure). The full details on the QW growth can be found in our previous work [19], [43]. By performing Hall measurements on Hall bars fabricated from the same QW wafer, we measured the charge density and the mobility to be $∼2.4\times 10^{11}$ cm^−2^ and $∼7.0\times 10^{6}$ cm^2^/V-s, respectively (at 2 K in the dark).

To control the intersubband absorption behavior of the TACIT device, the active region was enclosed with a symmetric dual-gate structure made of a pair of Ti/Au Schottky gates (Figure 1A). With the dual-gate structure, the charge density *n*_*s*_ of the QW and the perpendicular DC electric field *E*_*DC*_ can be set by applying electrostatic biases $V_{T\left(B\right)}$ applied to the top (bottom) gate; the charge density *n*_*s*_ is given by the sum voltage *V*_*T*_ + *V*_*B*_:

(1) $$n_{s}=n_{0}+\frac{c}{e}\left(V_{T}+V_{B}\right)$$

where *n*_0_ is the intrinsic charge density in the QW in the active region, and *c*/*e* is the charge tunability with unit voltage bias, with *c* being the geometric capacitance for the metal-2DEG structure per unit area and *e* the electron charge; the DC electric field is given by the difference voltage *V*_*T*_ − *V*_*B*_:

(2) $$E_{DC}=\frac{1}{d}\left(V_{T}-V_{B}\right)$$

where *d* is the separation distance between the top and bottom gates. In our Johnson noise measurements, the symmetric dual-gate structure enables the independent control of the charge density and the DC electric field (i.e. sweeping the DC electric field at a fixed charge density), allowing robust tuning of the intersubband absorption behavior of the QW while the Johnson noise of the TACIT device is being monitored.

**Figure 2:**
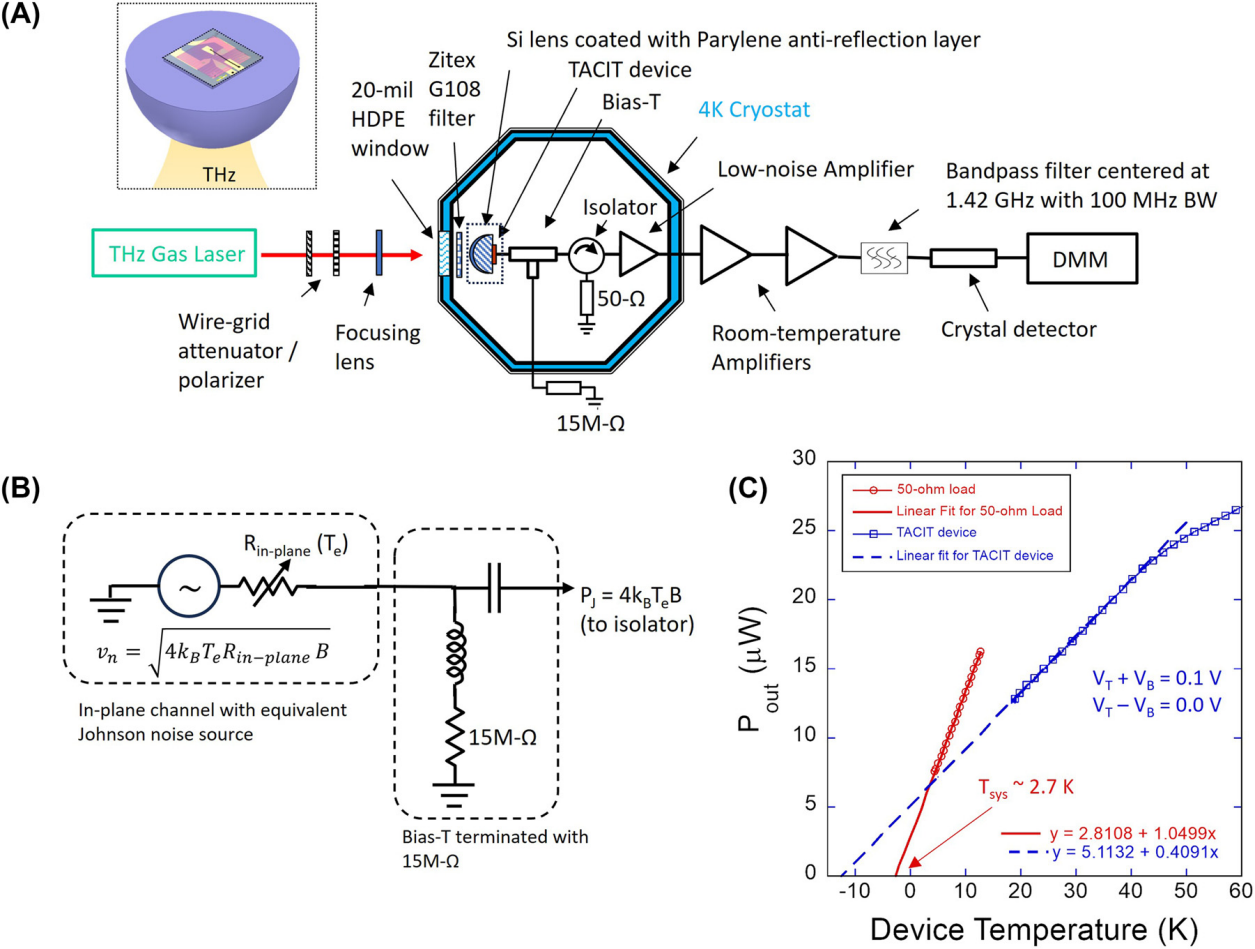
Experimental setup and calibration of system noise temperature. (A) Experimental setup for the Johnson noise measurement at 1.42 GHz. The inset shows the lens-antenna assembly for the TACIT device. (B) Equivalent circuit diagram for the in-plane channel of the device terminated with a 15-MΩ resistor when there is no DC current in the channel. (C) Calibration results for the system noise temperature (red circles) and the Johnson noise of the TACIT device (blue squares). The solid red line and the dashed blue line indicate linear fits.

For efficient coupling of THz radiation into the active region, the dual-gate structure of the active region was integrated with a half-wave bow-tie antenna (Figure 1B). In addition to concentrating the incoming THz electric fields into the active region, the antenna structure also orients their polarization to match the polarization required to satisfy the selection rule for intersubband absorption [30]. The design of the half-wave bow-tie antenna was optimized for 2.52 THz operation, with the overall length of the bow-tie antenna matching a half of the effective wavelength *λ*_eff_ at 2.52 THz at the air/Si interface ( $\lambda_{\text{eff}}/2=\lambda_{0}/2\sqrt{\left(\varepsilon_{Si}+1\right)/2}∼24\;\mu m$ where we have *λ*_0_ ∼ 119 μm for 2.5 THz and *ɛ*_*Si*_ = 11.7 for Si). Finite-element method (FEM) simulation of the antenna showed that the main lobe of its far-field pattern radiates mostly into the substrate within 30° angle of incidence relative to the optical axis (defined at the center of the active region along the QW growth direction), which is important for efficient coupling of THz radiation between the free space and the lens-antenna assembly. For impedance matching, the FEM simulation results showed that the antenna impedance (presented to the dual-gated active region) is 60 Ω + *i*10 Ω at 2.52 THz, which matches well to the impedance of the dual-gated active region $Z_{zz}=\left(50\text{ to }70\right)\Omega -i\left(0\text{ to }10\right)\Omega$ when the device is optimally tuned for 2.52 THz operation. The impedance *Z*_*zz*_ here is the impedance for the out-of-plane channel of the QW associated with the intersubband absorption and can be calculated using the impedance model for the intersubband [19], [22], [42] (see the equivalent circuit for the out-of-plane channel in the right side of Figure 2B for the case when the intersubband absorption frequency of the QW is matched to incoming THz frequency).

To measure the Johnson noise in the in-plane conduction channel of the device, a pair of Ohmic contacts (source and drain) was defined on the two ends of the ribbon-shaped 2DEG mesa (Figure 1B). The ribbon-shaped mesa was designed to allow wider (12-μm) Ohmic contacts to be used to reduce unwanted contact resistances in the in-plane resistance *R*_*in*−*plane*_. In contrast to the out-of-plane impedance *Z*_*zz*_ coupled to the antenna for THz coupling, the in-plane resistance *R*_*in*−*plane*_ is coupled to a 50-Ω load for the read-out of the Johnson noise at a microwave frequency (e.g. 1.42 GHz in this work). Since the in-plane resistance *R*_*in*−*plane*_ varies with the electron temperature *T*_*e*_ through the temperature dependence of in-plane mobility, the in-plane channel is modelled simply as a variable resistor *R*_*in*−*plane*_(*T*_*e*_), as shown in the circuit diagram at the bottom of Figure 1B. When there is no DC current in the in-plane channel, the output noise power of the in-plane channel at a microwave frequency is dominated by the Johnson noise power, which is directly proportional to the electron temperature [44], [45]. In this case, the in-plane channel can be represented with a Johnson noise source connected in series with a noiseless resistor (see Figure 2B) where the amplitude of the Johnson noise power is linearly proportional to the electron temperature sensitive to the intersubband absorption.

The fabricated TACIT device is shown in Figure 1C. We fabricated the required dual-gate structure using the indium-bond and stop-etch (IBASE) process that we developed to process both sides of high-mobility GaAs/Al_0.3_Ga_0.7_As thin films without significantly degrading its high mobility and charge density [43]. Figure 1C shows the 2 mm × 2 mm QW membrane (pink in color) where the TACIT device was defined at its center (see the inset). The QW membrane (less than 660-nm thick) is supported by a 3 mm × 3 mm high-resistivity Si substrate where Ti/Au electrodes were defined to electrically access the four terminals of the device (see the inset). The source, drain, and the bottom gate defined on the buried side of the QW membrane were bonded to the matching electrodes defined on the Si substrate with indium bonds (dotted squares) The top gate on the exposed surface of the QW membrane was routed to the upper part of the membrane and wire-bonded to the electrode on the Si substrate to avoid unwanted cross-over with the electrodes defined on the bonded side of the QW membrane. The rest of the bonded surface was filled with underfill epoxy to provide mechanical stability for the QW membrane. Further detail on the fabrication is provided in our previous work [19], [43].

After the fabrication, we performed DC measurements to characterize the temperature dependence of *R*_*in*−*plane*_ of the device (Figure 1D). The in-plane resistance was measured with a small ( <1μA ) DC current bias to avoid Joule heating, with the charge density and the perpendicular DC electric field fixed to constant values (*V*_*T*_ + *V*_*B*_ = 0.1 V and *V*_*T*_ − *V*_*B*_ = 0.0 V; see the inset in Figure 1C for the biasing scheme). The measured temperature dependence (red circles) was compared with that of the 2DEG sheet resistance (blue squares) measured from a Hall bar (with nominal channel width and length corresponding to 40 μm and 400 μm) fabricated from the same QW wafer. The temperature dependence shows that, with increasing temperature, *R*_*in*−*plane*_ increases non-linearly from 350 Ω (at 20 K) to 700 Ω (at 80 K), closely following the temperature dependence of the 2DEG sheet resistance. The relatively large resistance of the TACIT device compared to the 2DEG sheet resistance is attributed to the parasitic resistances present in the TACIT device, including both the contact resistances of the Ohmic contacts (∼250 Ω) and the parasitic resistance in the 2DEG extension outside the active region. The specific contact resistance was measured in a transfer line method (TLM) test device to be ∼1.5Ω⋅mm for each contact, and temperature-independent within tested temperature range (2 K–100 K). Lastly, to measure the tunability in the charge density *c*/*e* in Eq. (1), we measured the gate-voltage dependence of the charge density using a gated Hall bar processed in the same batch as the device, and found that we have the intrinsic charge density *n*_0_ ∼ 2.2 × 10^11^ cm^−2^ (slightly lower than the raw QW value after complete processing) and the charge tunability *c*/*e* ∼ 0.2 × 10^11^ cm^−2^/V.

### 2.2 Experimental setup for Johnson noise measurement

The experimental setup for the Johnson noise measurement is shown in Figure 2A. For quasi-optical coupling of THz radiation, the TACIT device was bonded on a high-resistivity Si lens (with a diameter of 10 mm) coated with Parylene anti-reflection coating (see the inset in Figure 2A). The device-lens assembly was then mounted in a copper mixer block, which was installed on the 4-K plate of a liquid helium cryostat. A 5-mil thick Mylar^®^ film was inserted between the mixer block and the 4-K plate to set the base temperature near 20 K. For temperature control, a resistive heater was installed on the mixer block. To suppress any unwanted DC current in the device (e.g., due to thermoelectric effects) that would generate additional noise (e.g. shot noise and thermal-fluctuation noise), the in-plane channel of the device was terminated with a 15-MΩ resistor through a bias-T (see the equivalent circuit in Figure 2B). In addition, no DC bias was applied to the in-plane channel of the device during the measurements. The electrostatic biases $V_{T\left(B\right)}$ to the top and bottom gates were provided by two DC voltage sources (see Figure 1C for the biasing scheme). To read out the Johnson noise power at a microwave frequency, the RF output of the bias-T was connected to a cryogenic high-gain, low-noise (*T*_*LNA*_ ∼ 1 K at 4 K) microwave amplifier (LNA) to amplify the Johnson noise power of the device above the noise contributions from the rest of the amplifier chain. To minimize the standing waves caused by the impedance mismatch between the TACIT device and the LNA, an isolator (an L-band microwave circulator terminated with a 50-Ω termination load) was inserted between the bias-T and the LNA. The bias-T, the isolator, and the LNA were all installed on the 4-K plate of the LHe cryostat.

Outside the cryostat, a series of room-temperature microwave amplifiers were used to further amplify the output noise power, with the nominal total gain of the system ∼80 dB. For the read-out of the Johnson noise, the amplifier output was filtered at room temperature with a band-pass filter centered at 1.42 GHz with a bandwidth of 100 MHz. The choice of the read-out frequency was based on the bandwidth of the system (1.35–1.75 GHz) limited by the isolator and the availability of the bandpass filter. The filtered output was then measured with a fast diode detector, which was later calibrated using an absolute power meter (Agilent E4418B).

To excite the THz intersubband transitions, we used a CO_2_-pumped far-infrared (FIR) molecular gas laser. Since the output of the gas laser was relatively high (∼50 mW for 2.52 THz, and ∼5 mW for 4.25 THz), we used a set of wire-grid polarizers to heavily attenuate the laser power. In addition, the second wire-grid polarizer was used to adjust the polarization of the attenuated beam to match the polarization that couples to the bow-tie antenna in the device. In TACIT mixers, the absorbed THz power can be roughly estimated by comparing the in-plane current-voltage curves (IVCs) of unpumped and pumped devices using the isotherm method originally developed for superconducting hot-electron bolometer (HEB) mixers [46], [47]. Based on this method, we estimate that the THz power absorbed in the device to be ∼3μ W at 2.52 THz when the device was tuned to optimal values of the charge density and the DC field (at *V*_*T*_ + *V*_*B*_ = 0.1 V and *V*_*T*_ − *V*_*B*_ = 0.0 V) for 2.52 THz operation.

To make sure that the noise of the amplifier chain is well below the expected Johnson noise of the device (with equivalent noise temperature >20 K at 20 K), we measured the noise temperature *T*_sys_ of our system using a variable-temperature 50-Ω load installed at the input of the bias-T. With the 50-Ω input that matches perfectly to the LNA, the noise power *P*_out_ measured at the output of the setup at 1.42 GHz is the sum of the Johnson noise of the 50-Ω load (with equivalent noise temperature *T*_load_ which is equal to its physical temperature for the Johnson noise) and the system noise:

(3) $$P_{\text{out}}=k_{B}G\left(T_{\text{load}}+T_{\text{sys}}\right)B$$

where *k*_*B*_ is the Boltzmann constant, *G* is the overall gain of the system (*G* ∼ 80 dB), and *B* is the frequency bandwidth (*B* = 100 MHz). By varying the physical temperature of the 50-Ω load and extrapolating the linear dependence of *P*_out_ on *T*_load_ at *P*_out_ = 0 (where we have *T*_load_ = −*T*_sys_), we measured *T*_sys_ ∼ 2.7 K (red circles and solid line in Figure 2C), which is slightly higher than the nominal input noise temperature of the LNA ( ∼1 K) due to the microwave losses in the isolator and the bias-T, but well below the expected Johnson noise temperature (>20 K) of the TACIT device.

To extract the electron temperature from the measured Johnson noise, its temperature dependence must be calibrated. For the calibration, we repeated the above calibration process with the input of the system replaced by the TACIT device tuned at *V*_*T*_ + *V*_*B*_ = 0.1 V and *V*_*T*_ − *V*_*B*_ = 0.0 V. In contrast to the 50-Ω load used for system noise calibration, the in-plane resistance *R*_*in*−*plane*_ of the TACIT device is typically not perfectly matched to 50 Ω, which results in the in-plane impedance matching efficiency $\alpha =\frac{4R_{\text{inplane}}R_{L}}{{\left(R_{in}+R_{L}\right)}^{2}}<1$ . The impedance mismatch causes the reflection of a portion of the device noise power at the LNA input. While the isolator (circulator terminated with a 50-Ω load) prevents the reflected noise power from getting transmitted in the rest of the amplifier chain, the small noise contribution from the 50-Ω termination load (from the isolator) shows up in the output noise power *P*_out_. With this additional noise contribution, the expression for *P*_out_ for the TACIT device is modified from Eq. (3) to

(4) $$P_{\text{out}}=k_{B}G\left[\alpha T_{e}+\left(1-\alpha \right)T_{50\;\Omega }+T_{\text{sys}}\right]B$$

where *T*_*e*_ is the electron temperature for the TACIT device (equal to its physical temperature in the absence of THz excitation and in-plane DC bias) and *T*_50 Ω_ = 4 K.

The measured calibration curve (blue squares in Figure 2C) shows the linear temperature dependence of *P*_out_ on *T*_*e*_ up to *T*_*e*_ ∼ 45 K and a slight roll-off at *T*_*e*_ > 45 K, indicating relatively constant *α* over a wide range of *T*_*e*_ (where Δ*P*_out_ ∼Δ*T*_*e*_). The slight roll-off is attributed to the increased device resistance at the higher temperature (Figure 1D). By comparing the slope of the linear fit for the device (blue dashed line) with the perfectly matched case for the 50-Ω load (red solid line), we estimate *α* ∼ 0.39 for the TACIT device tuned at *V*_*T*_ + *V*_*B*_ = 0.1 V and *V*_*T*_ − *V*_*B*_ = 0.0 V. This indicates that a significant portion (39 %) of the Johnson noise power of the device is coupled out in the output noise power *P*_out_ measured at 1.42 GHz. The measured value for *α* is roughly consistent with the calculated value of ∼0.43 based on the measured *R*_*in*−*plane*_ shown in Figure 1D. The small discrepancy is attributed to the non-negligible kinetic inductance of the TACIT device at 1.42 GHz and the microwave loss in the on-chip circuitry.

## 3 Experimental results and discussion

**Figure 3:**
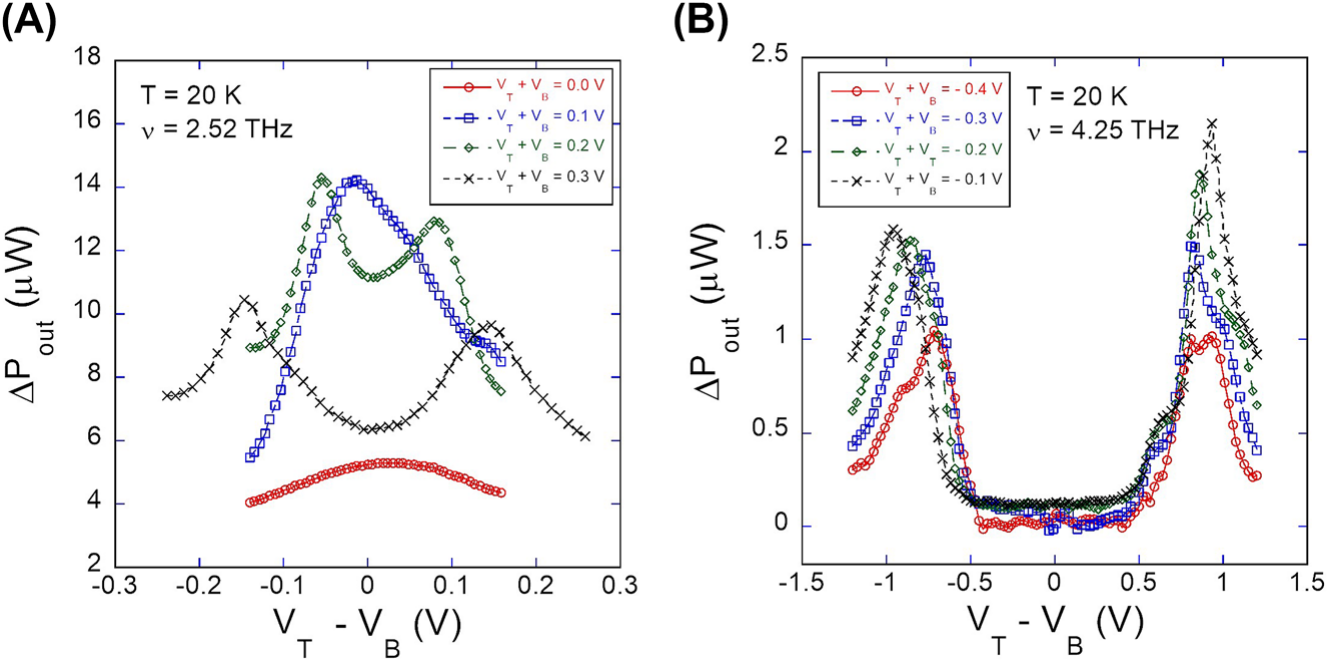
Johnson noise response at 20 K (a) at 2.52 THz and (b) at 4.25 THz.

The Johnson noise responses of the TACIT device to 2.52 THz and 4.25 THz measured at 20 K are presented in Figure 3. Each curve in the plots shows the DC field dependence (plotted in terms of the difference voltage *V*_*T*_ − *V*_*B*_) of the Johnson noise response measured at a fixed charge density (labelled in terms of the sum voltage *V*_*T*_ + *V*_*B*_). To account for experimental artifacts resulting from the small variations in the in-plane resistance *R*_*in*−*plane*_ and hence the impedance matching efficiency *α* at different DC field and charge density values, we measured the background Johnson noise of the device for each curve in the absence of THz excitation and plotted the difference (increase) in the Johnson noise power Δ*P*_out_ with THz excitation.

Distinct absorption features were observed at 2.52 THz and 4.25 THz. In the 2.52 THz measurements (Figure 3A), the Johnson noise response first emerged as a dispersed single peak at the lowest charge density (red curve at *V*_*T*_ + *V*_*B*_ = 0.0 V). With increasing charge density, the amplitude of the singe peak reached its maximum (blue curve at *V*_*T*_ + *V*_*B*_ = 0.1 V). With further increase in the charge density, the single-peak behavior evolved into a relatively symmetric double-peak behavior (green curve at *V*_*T*_ + *V*_*B*_ = 0.2 V). At the highest charge density, the amplitudes of the two peaks became smaller and their locations became separated farther apart (black curve at *V*_*T*_ + *V*_*B*_ = 0.3 V). At 2.52 THz, all the Johnson noise responses were observed near relatively small DC electric fields (set by $\left|V_{T}-V_{B}\right|\leq 0.3$ V), as expected from the intersubband absorption behavior of the 40-nm QW near its flat-band condition. In the measurements performed at smaller charge densities (where *V*_*T*_ + *V*_*B*_ < 0.0 V), relatively small or negligible increase in the Johnson noise was observed (not shown in Figure 3A).

In 4.25 THz measurements, we observed double-peak behavior in the Johnson noise response over the entire range of charge density values used for the measurements (−0.4 V ≤ *V*_*T*_ + *V*_*B*_ ≤ −0.1 V). Like the double-peak response observed in 2.52 THz measurements, the two peaks in the 4.25 THz responses became farther apart in DC field values with increasing charge density. However, in contrast to the 2.52 THz measurements, the Johnson noise responses to 4.25 THz were an order of magnitude smaller and occurred at higher magnitudes of the DC electric field ( $\left|V_{T}-V_{B}\right|>0.7$ V), as expected for the DC Stark-shifted intersubband absorption of the 40-nm QW far away from the flat band. At increased charge densities (where *V*_*T*_ + *V*_*B*_ > − 0.1 V), the response peaks started to occur at the DC field values near the threshold value (where $\left|V_{T}-V_{B}\right|∼1$ V), so the charge densities were set below this limit to avoid experimental artifacts from the possible gate breakdown [19].

**Figure 4:**
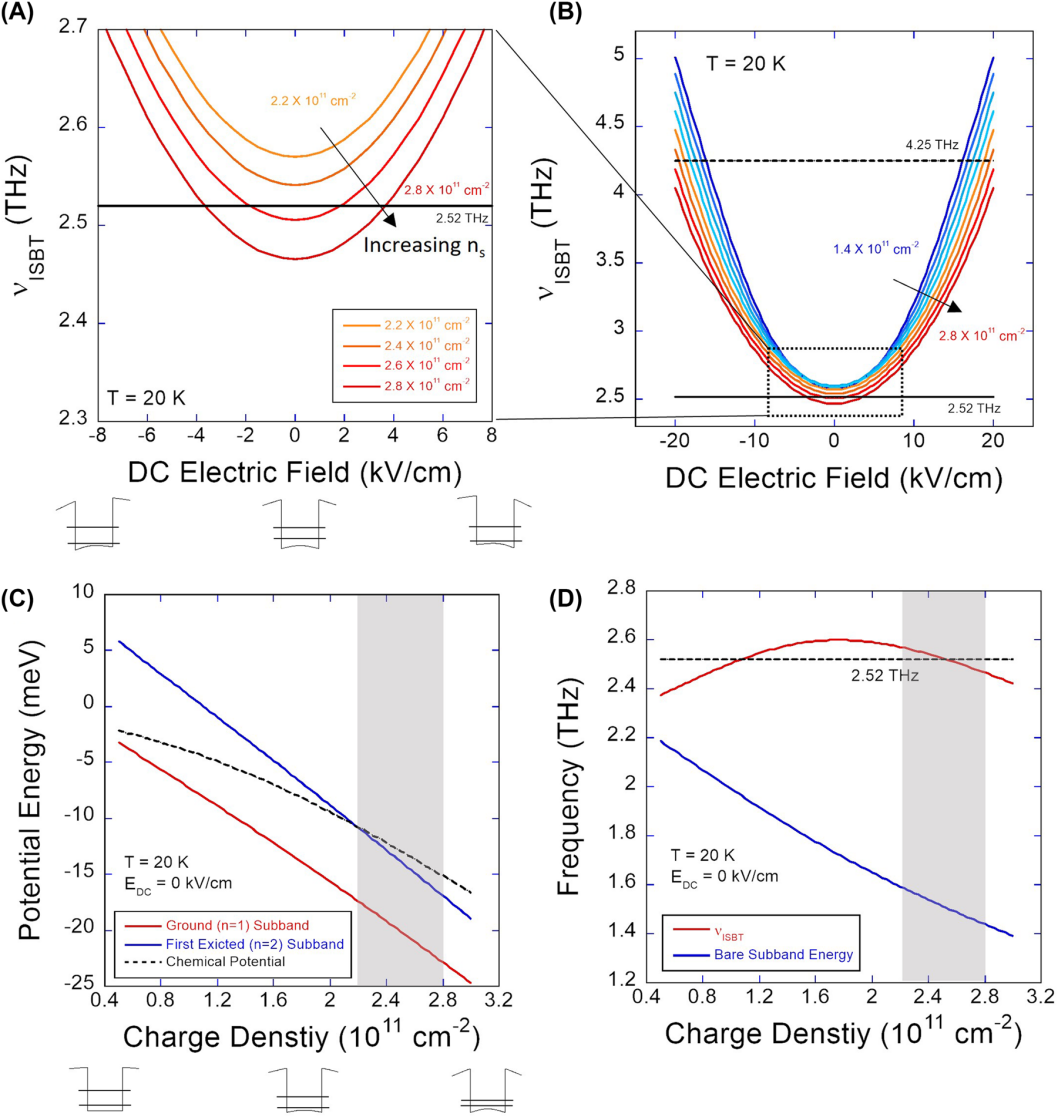
Calculated DC field dependence of intersubband absorption frequency at 20 K (A) near flat-band for selected charge densities and (B) for entire range of *E*_*DC*_ and *n*_*s*_. (C) Charge density dependence of the potential energy for the ground and the first excited subbands, and the chemical potential at *E*_*DC*_ = 0 kV/cm. The grey area shows the charge density range used for 2.52 THz measurements. (D) Charge density dependence of the intersubband absorption frequency and the bare subband spacing at *E*_*DC*_ = 0 kV/cm.

**Figure 5:**
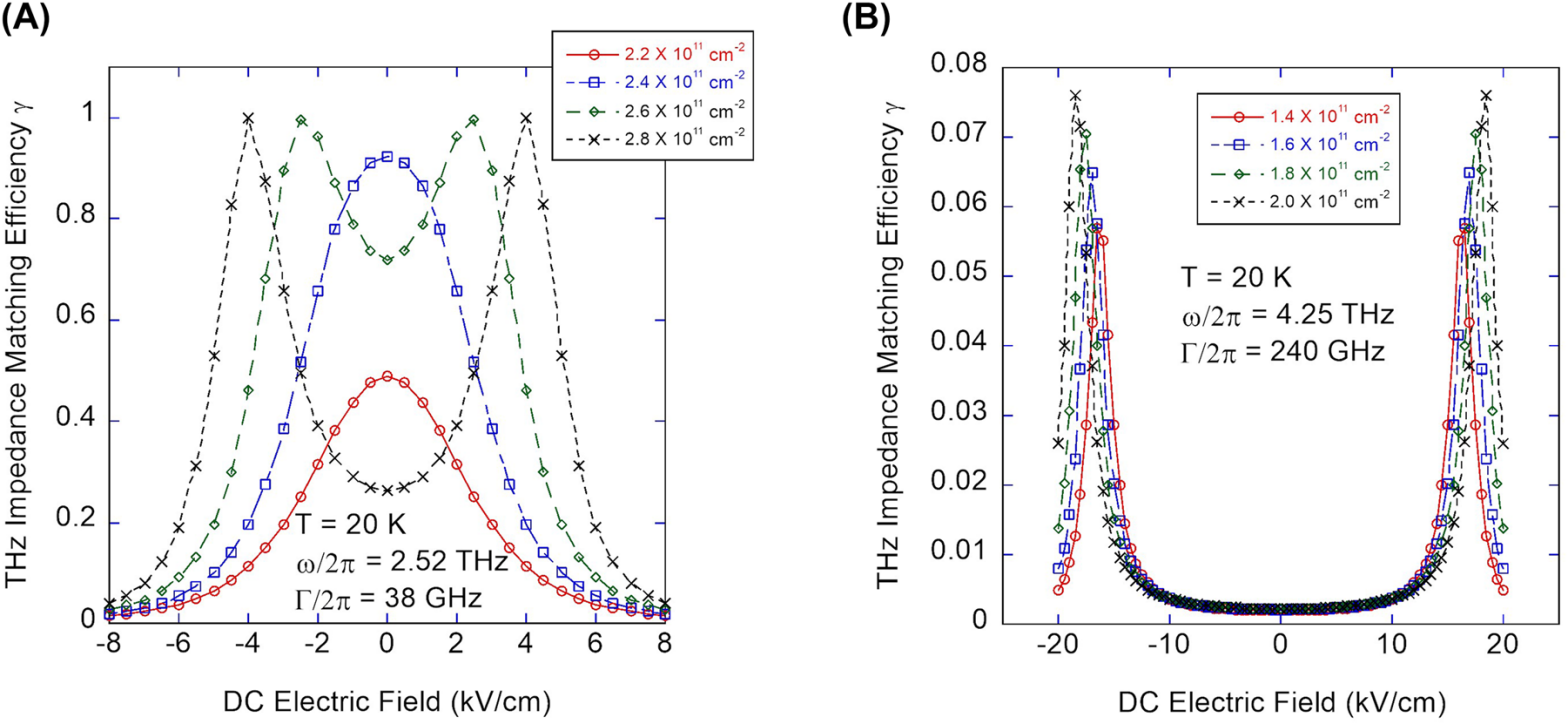
Simulated DC field response of the intersubband absorption at 20 K (A) for 2.52 THz and (B) for 4.25 THz.

To understand the observed Johnson noise behavior in terms of intersubband absorption, we calculated the 1 → 2 intersubband absorption frequency (Figure 4) and simulated the intersubband absorption response expected in the Johnson noise response (Figure 5). For the calculation of intersubband absorption frequency, we numerically solved the Schrödinger equation and Poisson’s equation self-consistently, considering the many-body effects on the subband energy (Hartree and exchange and correlation energy) and the collective effects on the absorption frequency (depolarization and exciton shifts). To simulate the intersubband response, the THz optical coupling efficiency was evaluated by calculating the impedance matching efficiency *γ* between the antenna and the dual-gated active region of the device using the standard expression for the power transmission:

(5) $$\gamma =\frac{4R_{\text{ant}}R_{zz}}{{\left(R_{\text{ant}}+R_{zz}\right)}^{2}+{\left(X_{\text{ant}}+X_{zz}\right)}^{2}}$$

where *Z*_ant_ = *R*_ant_ + *iX*_ant_ is the antenna impedance and *Z*_*zz*_ = *R*_*zz*_ + *iX*_*zz*_ is the impedance of the of the dual-gated active region.

As briefly discussed earlier, the out-of-plane impedance *Z*_*zz*_ can be calculated based on the impedance model for intersubband absorption [22], [42]: $Z_{zz}=\frac{1}{i\omega \varepsilon_{r}\varepsilon_{0}A}\left(d-\frac{\chi_{2\text{D}}\left(\omega \right)}{\varepsilon_{r}\varepsilon_{0}}\right)$ where *ɛ*_*r*_ is the relative permittivity for GaAs, *ɛ*_0_ the vacuum permittivity, *A* is the area of the dual-gated active region, *d* is the separation distance between the dual gates, and $\chi \left(\omega \right)$ is the 2D susceptibility of the 2DEG associated with the intersubband absorption [4], [33] given by

(6) $$\chi \left(\omega \right)=\frac{ef_{12}\left(N_{1}-N_{2}\right)}{m^{*}}\frac{1}{{\omega^{*}}^{2}-\omega^{2}+i2\omega \Gamma }$$

where *f*_12_ is the oscillator strength for the 1 → 2 intersubband transition, *N*_1_ − *N*_2_ the population difference between the ground subband and the first excited subband, m* the effective mass in GaAs, ω*/2*π* intersubband absorption frequency, and Γ/2π is the half-width half-maximum (HWHM) of the intersubband absorption resonance.

The results of the self-consistent calculation are shown in Figure 4. For the comparison with 2.52 THz measurements, the calculated DC field dependence of the intersubband absorption frequency near the flat band ( $\left|E_{DC}\right|\leq 8$ kV/cm) is plotted for the relevant charge densities (2.2 × 10^11^ cm^−2^ ≤ *n*_*s*_ ≤ 2.8 × 10^11^ cm^−2^) in Figure 4A. For 4.25 THz measurements, the full calculation results for the entire ranges of DC electric field ( $\left|E_{DC}\right|\leq 20$ kV/cm) and charge density (1.4 × 10^11^ cm^−2^ ≤ *n*_*s*_ ≤ 2.8 × 10^11^ cm^−2^) are shown in Figure 4B. For the calculation, the charge density values were converted from *V*_*T*_ + *V*_*B*_ in the experimental data using Eq. (1), but the DC electric field values were not directly converted from *V*_*T*_ − *V*_*B*_ due to the large discrepancy in the DC field calibration using Eq. (2). To match the experimental results, we used an effective QW width of 39 nm in our calculation [30], [48].

Before we compare the calculation results with the experimental data, we first comment on the effects of the DC electric field and the charge density on the intersubband absorption frequency. First, with increasing magnitude of the DC field |*E*_*DC*_|, the calculation results predict a blue shift in the absorption frequency over the entire range of charge density (Figure 4A and B). This is expected due to the DC Stark shift that increases the subband energy spacing by shifting down the ground (*n* = 1) subband to a lower potential energy [30], as depicted by the well diagrams below Figure 4A. Since the dependence of the DC Stark shift is quadratic in |*E*_*DC*_| near the flat band, the calculated absorption frequency curves in Figure 4A show symmetric, quasi-parabolic behavior. As shown in Figure 4B, the effect of the DC Stark shift can be substantial, accounting for the expected wide (2.5–5 THz) tunability in the absorption frequency in TACIT detectors.

With increasing charge density, a somewhat surprising red shift of the intersubband absorption is predicted over the entire range of DC electric field (Figure 4B). This red shift occurs even near the flat-band condition (Figure 4A) where one typically expects a blue shift due to depolarization shift [31]. We attribute this to the relatively high charge densities used in the experiments that start to significantly populate the second subband at *n*_*s*_ ≥ 2.2 × 10^11^ cm^−2^ near the flat band (Figure 4C). As the charge density increases, two competing effects determine the intersubband absorption frequency near the flat band. For one, the bare intersubband spacing – the energy required to move one electron from the ground to the excited subband at fixed momentum – decreases as electrons begin to accumulate at the edges of the well. These accumulated electrons result in an increase in the self-consistent Hartree potential at the center of the well (see QW diagrams below Figure 4C) that reduces the bare intersubband spacing (blue curve in Figure 4D), causing a red shift in the intersubband absorption frequency. For the other, the depolarization shift that always blue-shifts the intersubband absorption frequency remains approximately constant. Since the depolarization shift increases monotonically with the population difference between ground and excited subbands, the blue shift caused by the depolarization shift is typically greater than the red shift (decrease) of the bare intersubband spacing with increasing charge density if only the ground subband is occupied. However, once the second subband begins to get populated with increasing charge density, the population difference between the ground and the excited subbands remains approximately constant, resulting in a relatively constant depolarization shift with increasing charge density. This leads to a net red shift in the intersubband absorption frequency with increasing charge density beyond *n*_*s*_ ≥ 2.2 × 10^11^ cm^−2^. Similar red shifts with increasing charge density were predicted when the second subband starts to get significantly populated in the context of multi-subband plasmons [49], [50] (for ‘dark’ plasmons).

Compared with the experimental results, the calculated DC field and charge density dependence of the absorption frequency can qualitatively explain the observed Johnson noise response well. For 2.52 THz, the following qualitative description is possible: At *n*_*s*_ = 2.2 × 10^11^ cm^−2^ (top curve in Figure 4A), the intersubband absorption frequency is far above the laser line at 2.52 THz (solid black line in Figure 4A) over the entire range of DC field values. Near the flat band where the bottom of the curve is closest to the laser line, weak absorption is possible because of the finite value of the absorption resonance Γ, leading to a small single-peak absorption behavior observed in Figure 3A (red curve). At *n*_*s*_ = 2.4 × 10^11^ cm^−2^, the red shift brings the entire absorption frequency curve down closer to the laser line but without crossing it, resulting in the stronger single-peak absorption (blue curve in Figure 3A). At *n*_*s*_ = 2.6 × 10^11^ cm^−2^, the further red-shifted absorption frequency curve starts to intersect the laser line at two DC electric field values at which the intersubband absorption frequency of the QW is matched 2.52 THz. This leads to the observed symmetric double-peak behavior (green curve in Figure 3A). Finally, at *n*_*s*_ = 2.8 × 10^11^ cm^−2^, the absorption frequency curve crosses the laser line at higher $\left|E_{DC}\right|$ values, resulting in the similar double-peak behavior but with the two peaks now separated farther apart (black curve in Figure 3A). In contrast to the 2.52 THz results, the double-peak absorption behavior is expected to be observed at all charge density values (1.4 × 10^11^ cm^−2^ ≤ *n*_*s*_ ≤ 2.8 × 10^11^ cm^−2^) for the 4.25 THz measurements, as the laser line for 4.25 THz (dashed black curve in Figure 4B) always intersects the absorption frequency curves at two *E*_*DC*_ values far away from the flat band (Figure 4B). With increasing charge density, the predicted red shift moves the entire curves down, resulting in the observed outward shifts of the two absorption peaks in Figure 3B.

For more direct comparison between the calculation results and the experimental data, the DC field dependence of the impedance matching efficiency *γ* was calculating based on Eq. (5) and plotted in Figure 5. For the antenna impedance *Z*_ant_, we used the impedance values from the FEM simulation results: *Z*_ant_ = 60 Ω + *i*10 Ω for 2.52 THz and *Z*_ant_ = 70 Ω − *i*25 Ω for 4.25 THz. For the calculation of the device impedance *Z*_*zz*_, the necessary QW parameters (*f*_12_, *N*_1_ − *N*_2_, and ω*) in Eq. (6) were acquired from the self-consistent calculation. For the HWHM (Γ/2*π*), we used a constant value of 38 GHz for 2.52 THz and 240 GHz for 4.25 THz. The large discrepancy in the linewidth values is due to its expected DC field dependence caused by increased interface roughness scattering when the electrons in the QW are pushed closer to the surfaces of the QW sample with the higher $\left|E_{DC}\right|$ [31]. The linewidth values used for the calculation are roughly consistent with the experimental values measured in a similar modulation-doped 40-nm square QW structure at 2 K [31].

The simulated intersubband responses at 2.52 THz and 4.25 THz successfully reproduced the absorption features observed in the Johnson noise responses (Figure 5). At 2.52 THz, the model replicates the transition from a single-peak to a double-peak response with increasing charge density observed in the Johnson noise response. At 4.25 THz, the simulated responses reproduce the observed double-peak response with the peaks shifting outward and the peak amplitude increasing with increasing charge density. In addition, the order-of-magnitude smaller responses observed at 4.25 THz compared to 2.52 THz responses is predicted well in the model.

While the agreement between the simulation and the experiment is reasonably good, a more physically accurate and comprehensive model is desirable to fully simulate the observed absorption behavior. Such a model would account for the effects of the changes in the linewidth more dynamically using a more sophisticated theory [51]. In addition, the model would include the effect of the electron heating on the intersubband absorption. If the electron temperature is increased due to the intersubband absorption, then the increased electron temperature would in turn affect the intersubband absorption efficiency. This temperature effect is predicted to be dependent on the DC electric field and, along with the linewidth effect, may contribute to more fine absorption features (e.g. slight asymmetry observed in the double-peak responses at 2.52 THz and 4.25 THz in Figure 3, decreased peak amplitude for the 2.52 THz responses at *V*_*T*_ + *V*_*B*_ = 0.3 V in Figure 3A, etc.) that are not accurately simulated in our current model.

**Figure 6:**
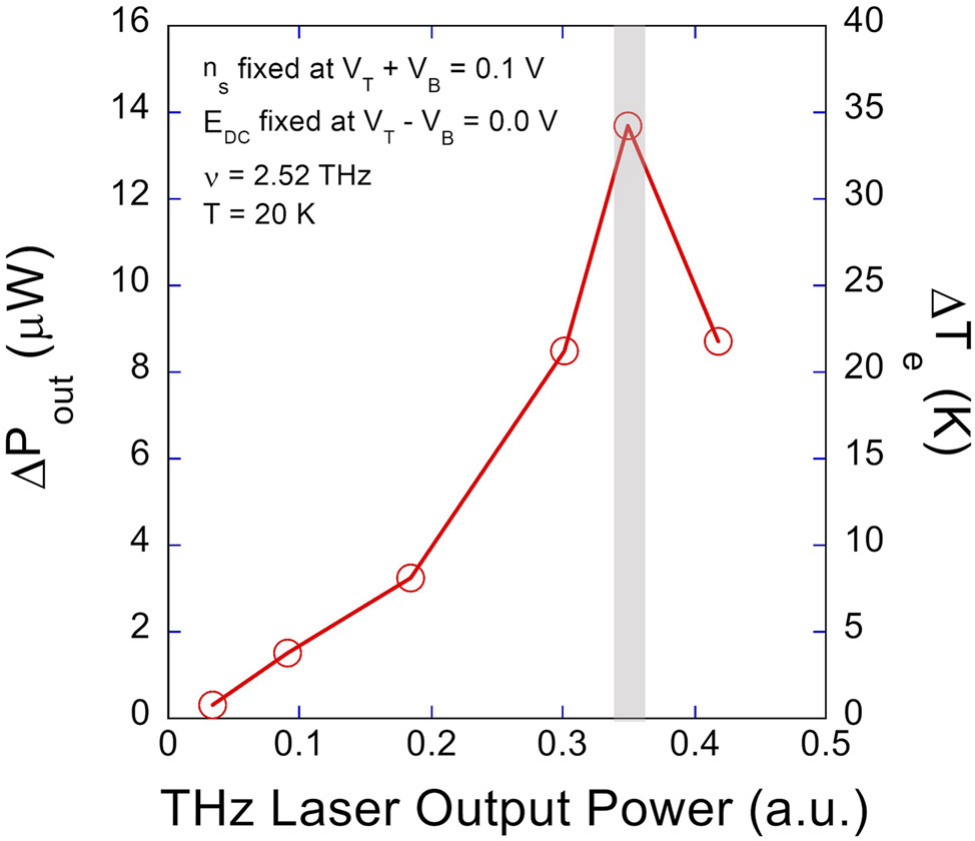
Rough power dependence of the Johnson noise response to 2.52 THz at 20 K when the device is optimally tuned at *V*_*T*_ + *V*_*B*_ = 0.1 V and *V*_*T*_ − *V*_*B*_ = 0.0 V at 20 K. The grey bar indicates the THz power used for the 2.52 THz measurements shown in Figure 3A.

Finally, we extracted the increase in the electron temperature from the Johnson noise response when the device was optimally tuned for 2.52 THz operation (at *V*_*T*_ + *V*_*B*_ = 0.1 V and *V*_*T*_ − *V*_*B*_ = 0.0 V; at the peak of the Johnson noise response in the blue curve in Figure 3A). For the calibration, we used the temperature dependence of the Johnson noise power acquired at the same operating point of the device (Figure 2C). The calibrated Johnson noise response as well as its rough dependence on THz power at 2.52 THz is shown in Figure 6. The THz power used for the measurements in Figure 3A is indicated with the grey bar. The power dependence shows that the increase in the electron temperature reaches its maximum of ∼35 K when the maximum THz absorption occurs in the device. In addition, the non-monotonic dependence suggests that the intersubband absorption efficiency is significantly affected by the THz power (intensity) that must be optimized on top of the DC electric field and charge density for the maximum intersubband absorption. This power (intensity) dependence is likely due to the possible intensity-dependent shifts in the intersubband absorption frequency [32] and saturation effects, and will be more thoroughly investigated in our future studies. Such a study would be important both for a more in-depth understanding of the intensity dependence and the saturation phenomena in THz intersubband absorption [52], [53] and for practical device engineering of TACIT mixers, especially in the perspective of optimizing the THz local-oscillator power for low-noise mixing operation.

Before we conclude, we highlight that the Johnson noise measurement allowed the study of intersubband absorption of a small (∼20,000) number of electrons in the single TACIT device. With further reduction in the size of the active region and modification of the antenna structure (e.g. by implementing a resonator structure [54], [55], [56], [57]), this approach may open up an interesting possibility of electrically probing the intersubband absorption behavior of even fewer electrons, especially in the ultrastrong coupling regime. Furthermore, relatively simple implementation of the technique and the capability to directly measure the electron temperature, coupled with robust tunability that can be achieved in TACIT-like device structure, make the Johnson noise thermometry a useful tool for probing intersubband absorption in diverse QW systems at different physical conditions (charge density, DC electric field, temperature, THz intensity, etc.), which is useful both for fundamental spectroscopic study of intersubband absorption and for device engineering of various practical intersubband devices.

## 4 Conclusions

In conclusion, we have investigated the THz intersubband absorption behavior of a single, modulation-doped, 40-nm wide GaAs/AlGaAs square QW using the Johnson noise thermometry. By leveraging the in-plane read-out structure and the tunability in the TACIT device, we measured the Johnson noise responses to 2.52 THz and 4.25 THz at 20 K as a function of the DC electric field and the charge density. By self-consistently calculating the intersubband absorption frequency and by simulating the expected intersubband responses using the impedance model for intersubband absorption, we showed that the observed Johnson noise responses can be explained well in terms of the DC-Stark-shifted intersubband absorption behavior near the flat band (for 2.52 THz) and far away from the flat band (for 4.25 THz). Finally, by calibrating the temperature dependence of the Johnson noise when the device is optimally tuned for 2.52 THz operation, we deduced an increase in the electron temperature Δ*T*_*e*_ ∼ 35 K when the maximum absorption of THz power ( ∼3μ W) occurs in the device. Relatively simple but sensitive microwave measurements and the capability to directly measure the electron temperature resulting from intersubband absorption make the Johnson noise thermometry useful for both device engineering of practical intersubband devices and for fundamental study of intersubband physics.
